# Unraveling Metabolic Syndrome in Youth: The Obesity Epidemic’s Hidden Complication

**DOI:** 10.3390/children12040482

**Published:** 2025-04-08

**Authors:** Dana-Teodora Anton-Păduraru, Dana Elena Mindru, Raluca Stefania Stănescu, Felicia Trofin, Claudiu Cobuz, Maricela Cobuz, Lucia Maria Sur, Antoneta Petroaie, Ana Maria Slănină, Mihaela Manole, Ana Simona Bocec, Adriana Cosmescu

**Affiliations:** 1 Department of Mother and Child Medicine, “Grigore T. Popa” University of Medicine and Pharmacy, 700115 Iasi, Romania; dana.anton@umfiasi.ro (D.-T.A.-P.); mindru.dana@umfiasi.ro (D.E.M.); simona.drochioi@umfiasi.ro (A.S.B.); 2 “Sf. Maria” Children Emergency Hospital, 700309 Iasi, Romania; 3 Department of Morpho-Functional Sciences II, “Grigore T. Popa” University of Medicine and Pharmacy, 700115 Iasi, Romania; raluca.stanescu@umfiasi.ro; 4 Microbiology—Department of Preventive Medicine and Interdisciplinarity, “Grigore T. Popa” University of Medicine and Pharmacy, 700115 Iasi, Romania; 5 “Sf. Spiridon” County Emergency Clinical Hospital, 700111 Iasi, Romania; 6 Department of Biomedical Sciences, Faculty of Medicine and Biological Sciences, “Stefan cel Mare” University, 720229 Suceava, Romania; claudiu.cobuz@usm.ro; 7 “Sfântul Ioan cel Nou” Emergency County Clinical Hospital, 720224 Suceava, Romania; cobuz.maricela@spjsv.ro; 8 Department of Child and Mother, “Iuliu Hatieganu” University of Medicine and Pharmacy, 400347 Cluj-Napoca, Romania; sur.maria@umfcluj.ro; 9 Family Medicine—Department of Preventive Medicine and Interdisciplinarity, “Grigore T. Popa” University of Medicine and Pharmacy, 700115 Iasi, Romania; antoneta.petroaie@umfiasi.ro (A.P.); ana.slanina@umfiasi.ro (A.M.S.); mihaela.manole@umfiasi.ro (M.M.); adriana.cosmescu@umfiasi.ro (A.C.)

**Keywords:** obesity, complications, metabolic syndrome, children, adolescents

## Abstract

Background/Objectives: One of the metabolic complications of obesity is known as Metabolic Syndrome (MetS). This narrative review aims to synthesize current research on MetS in young populations, identify knowledge gaps, and guide future studies and funding priorities. It provides evidence-based insights into screening, diagnosis, and treatment, offering practical guidance for healthcare professionals. Methods: A comprehensive search of the literature was conducted to identify relevant studies on obesity in MetS in PubMed and Google Academic electronic database. The search was performed using a combination of “obesity”, “complications”, “metabolic syndrome”, “children”, and “adolescents” keywords. Studies were selected based on predefined inclusion and exclusion criteria to ensure relevance and methodological rigor. Results: The lack of universally accepted diagnostic criteria for MetS in children has led to inconsistencies in its definition across medical literature. Irrespective of the chosen diagnostic approach, the prevalence of MetS in children and adolescents has exhibited a concurrent rise with the increasing rates of obesity in this age group. The pathophysiology of MetS remains incompletely understood, with ongoing discussions on the interplay of genetic, epigenetic, environmental, dietary, and lifestyle factors. Screening for MetS is recommended for overweight and obese children. Conclusions: Establishing global, consensus-driven criteria that account for ethnicity, gender, and age would enhance diagnostic accuracy and treatment approaches. The prevention of excessive weight among children and adolescents stands as a paramount objective within modern society’s healthcare system. Considering the complexity of the disease and the treatment, the team must be multidisciplinary.

## 1. Introduction

Rising global obesity rates led the World Health Organization (WHO) to coin the term “globesity” in 2001, combining “global” and “obesity” [1]. By 2025, an estimated 268 million school-aged children will be overweight, with 91 million classified as obese [2]. This increase is strongly associated with a higher risk of cardiovascular disease and comorbidities such as hypertension (HTN), hyperinsulinemia, type 2 diabetes mellitus (T2DM), dyslipidemia, and non-alcoholic fatty liver disease (NAFLD), with over half of obese children experiencing multiple complications [3,4].

MetS is a significant public health issue that can persist into adulthood. It involves metabolic disturbances that contribute to atherosclerotic cardiovascular disease (CVD), T2DM, and vascular and neurological complications [5,6,7]. MetS is also a major risk factor for heart failure with preserved ejection fraction (HFpEF), which increases hospitalization and mortality risk [8].

First identified about four decades ago, MetS has been known by various names, including “syndrome X”, “insulin-resistant syndrome”, and “Reaven syndrome”; Gerald M. Reaven introduced the term “Syndrome X” in 1988 [6,9].

### Motivation and Aim of the Narrative Review

The increasing prevalence of MetS among children and adolescents poses a major public health challenge, emphasizing the need for a comprehensive review. The rising obesity rates in this age group are linked to long-term health risks, including atherosclerosis and cardiovascular conditions. Understanding the underlying mechanisms, particularly the role of central obesity, is crucial for effective prevention and management [10].

This narrative review aims to synthesize current research on MetS in young populations, identify knowledge gaps, and guide future studies and funding priorities. It provides evidence-based insights into screening, diagnosis, and treatment, offering practical guidance for healthcare professionals, including paediatricians and endocrinologists. By examining the pathophysiology, risk factors, and long-term consequences of MetS, the review explores biological, environmental, and behavioural contributors to its development.

Additionally, it highlights gaps in the literature, proposes targeted prevention and intervention strategies, and addresses the public health implications of MetS. Ultimately, this analysis seeks to enhance care quality and outcomes for affected children and adolescents while informing more effective clinical and policy approaches.

## 2. Materials and Methods

A comprehensive search of the literature was conducted to identify relevant studies on obesity in MetS in PubMed and Google Academic electronic database. The search was performed using a combination of “obesity”, “complications”, “metabolic syndrome”, “children”, and “adolescents” keywords and Boolean operators. Additional searches were conducted using reference lists of selected articles to ensure thorough coverage of relevant studies.

Selection criteria included alignment with the research question, “Is metabolic syndrome a complication of obesity?” as well as publication year, study type, and quality of findings. Studies were selected based on predefined inclusion and exclusion criteria to ensure relevance and methodological rigor.

The inclusion criteria were: only peer-reviewed journal articles; cohort studies, systematic reviews studies or metaanalyses; articles that provide original data or systematic reviews/meta-analyses; published between 2015–2025; research focusing on humans, obesity, children or adolescents (0–18 years), specific MetS components like insulin resistance, HTN, and dyslipidemia; articles available in English; articles reporting on definition, prevalence, indices, oxidative stress, adipocytokines, renin-angiotensin system, epigenetics, gut microbiota, screening; research with clearly defined sample populations; longitudinal or cross-sectional studies that provide empirical data.

The exclusion criteria were: non-peer-reviewed sources such as editorials, commentaries, opinion pieces, conference papers, letters to the editor, blogs, and government reports; studies with incomplete or unclear methodologies; duplicates of previously included studies; articles published before 2015, unless historically relevant; non-English studies; studies focusing only on animal models or in vitro experiments; research on obesity or MetS in rare genetic disorders rather than general populations; studies that do not differentiate between obesity-related MetS and other metabolic disorders; articles focusing on non-human subjects or non-relevant populations (adults); studies without clear diagnostic criteria for obesity or MetS; research with small sample sizes or low statistical power; trials with unclear or poorly defined interventions; studies lacking control groups or comparison data; research that does not measure long-term metabolic outcomes. Articles were excluded if they focused on adults, addressed non-obesity-related causes of MetS, used animal models, or lacked specific MetS components like insulin resistance (IR), HTN, and dyslipidemia. Studies on unrelated populations (e.g., athletes, youth with eating disorders) and non-peer-reviewed sources were omitted. Research that grouped youth and adults without distinguishing age groups or lacked key MetS markers was also excluded. Additionally, studies published before obesity became a significant public health issue were deemed outdated.

Selected articles underwent structured categorization and synthesis, ensuring a comprehensive review of MetS in obese youth. Following initial searches, articles were excluded based on duplication, publication date, and title relevance. A subsequent abstract screening led to further exclusions due to relevance, accessibility, or topic misalignment. A full-text review then eliminated some articles due to methodological inconsistencies, quality concerns, or language barriers.

Data from eligible studies were extracted using a structured template, including details on authors and publication year. Extracted data were categorized thematically to identify trends, gaps, and inconsistencies within the existing literature. A qualitative synthesis was performed to summarize findings. To ensure the reliability and validity of included studies, a manual quality assessment was conducted. Studies meeting high-quality criteria were given greater emphasis in the review, while potential biases and limitations were acknowledged.

## 3. Results

### 3.1. Definition of MetS in Children and Adolescents

The lack of universally accepted diagnostic criteria for MetS in children has led to inconsistencies in its definition across medical literature [10,11]. While established criteria exist for adults, their applicability to children is debated due to developmental factors, including puberty-related hormonal changes and changes in body proportions [11,12,13]. The American Heart Association (AHA) highlighted these limitations in 2009, emphasizing the need for consistent and demographically applicable diagnostic criteria. In adults, MetS diagnosis remains stable in about 75% of cases over three years, whereas in children, it is only 31.6% in the short term and 45.5% upon long-term re-evaluation [14].

MetS is a cluster of cardiometabolic risk factors primarily characterized by central obesity, indicated by elevated waist circumference (WC), along with IR, HTN, elevated triglycerides (TG), and low levels of high-density lipoproteins (HDL) [15]. WC serves as a key diagnostic criterion due to its strong association with IR, lipid profiles, and blood pressure (BP). Research indicates that children with WC above the 90th percentile face significantly higher cardiovascular risk [16].

Various organizations have proposed definitions of paediatric MetS [17,18]. The WHO (1998) defined it with IR as a central component, requiring its presence along with at least two other criteria, such as HTN, dyslipidaemia, obesity, or microalbuminuria [2]. The European Group for the Study of Insulin Resistance (EGIR) (1999) emphasized abdominal obesity but replaced microalbuminuria with hyperinsulinemia [19]. The National Cholesterol Education Program—Adult Treatment Panel III (NCEP-ATP III) (2001) defined MetS based on three or more of the following:

abdominal obesity (WC ≥ 90th percentile), TG ≥ 110 mg/dL, HDL ≤ 40 mg/dL

HTN (BP ≥ 90th percentile), and fasting plasma glucose (FPG) ≥ 110 mg/dL [20,21].

Cook et al. (2003) and de Ferranti et al. (2004) proposed similar diagnostic frameworks with variations in threshold values [22,23]. The International Diabetes Federation (IDF) (2007) introduced paediatric-specific criteria, defining MetS for ages 10–16 based on central obesity and at least two additional metabolic abnormalities [6,16,24]. The Turkish Society of Endocrinology and Metabolism (2009) also developed MetS guidelines, incorporating obesity, IR, and cardiometabolic factors [9].

The Identification and Prevention of Dietary- and Lifestyle-Induced Health Effects in Children and Infants (IDEFICS) study (2014) provided new age- and sex-specific criteria for prepubertal children across Europe but did not quantify disease risk [25]. Zong et al. (2022) proposed an international MetS definition for ages 6–17, requiring WC ≥ 90th percentile and at least two additional features, such as TG, low HDL, HTN, or impaired FPG [26].

A comparative analysis of these definitions is presented in Table 1.

Noubiap et al. (2022) emphasize the need for standardized MetS diagnostic criteria to improve global monitoring and prevention. Their proposed refinements aim to enhance diagnostic accuracy, enabling earlier and more effective interventions. This review serves as a foundation for future research on developing robust paediatric MetS diagnostic frameworks [27].

### 3.2. Prevalence of MetS

Determining the precise prevalence of MetS in paediatric populations is challenging due to the lack of a standardized definition. Reported prevalence rates range from 6% to 39%, varying based on diagnostic criteria, age, gender, BMI, socioeconomic status, sedentary behaviour, and ethnicity [3,6,7,9,16,28]. Regardless of the criteria used, MetS prevalence has increased alongside rising childhood obesity rates. Frías et al. (2023) estimate that approximately one-fourth of the global population is affected by MetS [7].

A 2016 UNICEF report noted a rise in MetS prevalence among children and adolescents in Western Europe and the U.S., increasing from 2% to 25% since the mid-1990s [9]. The Bogalusa study reported a prevalence of 4% in Caucasian children and 3% in African-American children [29]. A meta-analysis by Reisinger et al. (2021) found a wide range of MetS prevalence in children, from 0.3% to 26.4% [16]. Similarly, Friend et al. (2013) observed a prevalence of 11.9% in overweight children and 29.2% in obese individuals [30]. Among adolescent girls with polycystic ovary syndrome (PCOS), MetS prevalence ranged from 4.08% to 60.78% [31]. According to the WHO (2017), about 70% of individuals with diabetes mellitus (DM) develop MetS during puberty [32].

Different diagnostic criteria yield varying prevalence rates:

- The IDF criteria report a prevalence of 0.3% to 9.5%, lower than Ferranti et al.’s [23] definition (4.0% to 26.4%) [16].
- A meta-analysis by Bitew et al. (2020) found MetS prevalence of 24.1% (IDF) and 56.32% (de Ferranti et al.) [23], significantly higher than in the general population (3.98% and 8.19%, respectively) [33].
- A review of 49 studies showed prevalence rates of 3.70% (IDF), 5.40% (ATP III), 14.78% (de Ferranti et al.) [23], and 3.90% (WHO) [11].

On the other hand, given the increasing prevalence of obesity in children and adolescents, the prevalence of MetS will also increase alarmingly.

Regional variations in MetS prevalence are evident, with higher rates in Asian populations, potentially due to physiological traits such as increased abdominal adiposity, dietary patterns high in refined carbohydrates and saturated fats, and genetic predispositions, including apolipoprotein A1 (APOA1) polymorphisms [34,35,36]—Table 2.

**Figure 1:**
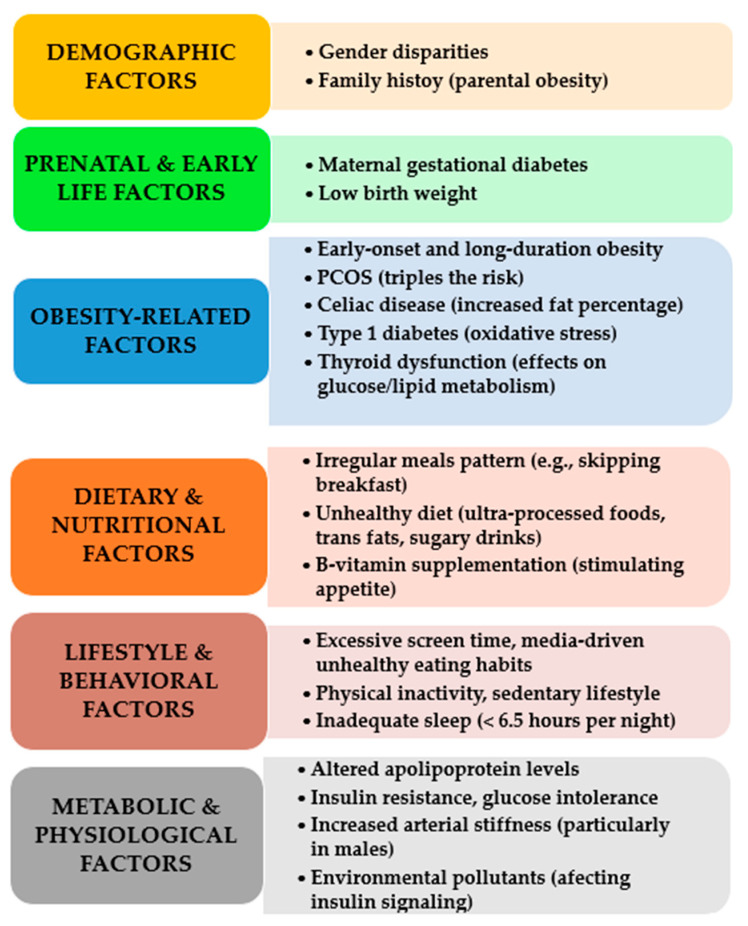
Key risk factors for pediatric MetS.

The primary risk factors for paediatric MetS were identified through various studies, systematically categorized, synthesized, and are presented in Figure 1 [7,11,31,40,63,64,65,66,67,68,69,70,71].

MetS and was associated with a 61% lower incidence of MetS overall [72]. A systematic review and meta-analysis by Ulloque-Badaracco et al. (2023) found that higher vitamin B12 levels were inversely associated with MetS [73]. The study of Jayashri et al. (2018) demonstrated that vitamin B12 deficiency was associated with the severity of glucose tolerance, a key component of MetS, supporting the inverse relationship between vitamin B12 levels and MetS risk [74]. Regarding vitamin C, a study by Wong et al. (2020) found an inverse relationship between serum vitamin C levels and MetS [75]. The study by Vitezova et al. (2015) found that higher serum vitamin D levels were associated with a lower prevalence of MetS and a more favorable metabolic profile [76]. Shi et al. (2023) found an inverse relationship between MetS and vitamin B12, C, and D intake, while Vajdi et al. (2020) highlighted the protective effects of fibre, whole grains, fruits, vegetables, vitamin E, niacin, magnesium, potassium, linoleic acid, and docohexaenoic acid (DHA) [77,78]. Thereafter, these studies suggest that higher intakes and serum levels of vitamins B12, C, and D are associated with a lower risk of MetS. Genetics further contribute to MetS risk. Nagrani et al. (2023) and Bokhari et al. (2018) identified obesity- and lipid-related genes as primary drivers of genetic predisposition in MetS cases [68,79].

Mets is highly influenced by modifiable factors such as dietary choices, food composition, and lifestyle behaviours. By adopting a nutrient-dense diet, maintaining an active lifestyle, managing stress, and avoiding harmful habits, individuals can significantly reduce their risk of MetS and its associated complications.

MetS is a collection of interrelated metabolic abnormalities that substantially elevate the risk of cardiovascular diseases, type 2 diabetes, and cerebrovascular disorders. The primary components of MetS include central obesity, insulin resistance, dyslipidaemia, hypertension, and a pro-inflammatory state. Several modifiable factors, including dietary habits, the nutritional composition of food, and lifestyle choices, play a pivotal role in both the prevention and management of MetS.

(1) Dietary Habits and Their Impact on MetS

Poor dietary patterns are significant contributors to the development and progression of MetS. Modifications in dietary intake can mitigate associated risks and improve overall metabolic health.

(2) Unhealthy Dietary Patterns That Exacerbate MetS

High consumption of refined carbohydrates such as white bread, pasta, and sugary foods increases insulin resistance and blood glucose levels. Excessive intake of saturated and trans fats from sources like fried foods and processed meats contributes to dyslipidaemia and cardiovascular disease. Overconsumption of sugar-sweetened beverages leads to obesity, increased triglyceride levels, and insulin resistance. Irregular meal patterns or frequent meal skipping disrupt metabolic homeostasis and exacerbate glucose dysregulation.

(3) Dietary Modifications to Improve MetS

Adoption of the Mediterranean Diet, which is rich in olive oil, nuts, fish, and whole grains, reduces systemic inflammation and enhances insulin sensitivity. Implementation of the DASH Diet, or Dietary Approaches to Stop Hypertension, lowers blood pressure and improves lipid profiles. Consumption of low glycemic index foods such as legumes, whole grains, and non-starchy vegetables enhances insulin function and stabilizes blood glucose levels. Increased dietary fibre intake from sources like fruits, vegetables, and whole grains aids in weight management and improves lipid metabolism.

(4) Nutritional Components and Their Influence on MetS

Certain nutrients and food constituents can either exacerbate or mitigate MetS progression.

- Nutrients That Contribute to MetS:

Excess sodium intake, particularly from processed foods, increases blood pressure and fluid retention. Added sugars and artificial sweeteners promote obesity, insulin resistance, and metabolic dysfunction. Saturated and trans fats elevate low-density lipoprotein cholesterol and heighten cardiovascular risk.

- Nutrients That Offer Protective Effects Against MetS:

Omega-3 fatty acids, found in fish, flaxseeds, and walnuts, reduce systemic inflammation and lower triglyceride levels. Polyphenols and antioxidants, present in berries, green tea, and dark chocolate, protect against oxidative stress and improve vascular function. Probiotics and prebiotics, sourced from yogurt, kefir, and fermented foods, enhance gut microbiota composition and support metabolic health. Magnesium and potassium, abundant in avocados, bananas, and leafy greens, regulate blood pressure and support cardiovascular function.

(5) Lifestyle Factors and Their Role in MetS

Beyond dietary considerations, lifestyle behaviours significantly influence the onset and progression of MetS. Physical inactivity leads to weight gain, increased insulin resistance, and a higher risk of cardiovascular disease. Sleep deprivation or poor sleep quality disrupts metabolic homeostasis and elevates hunger-related hormones, contributing to weight gain. Chronic psychological stress increases cortisol levels, leading to central obesity and impaired insulin regulation. Tobacco use and excessive alcohol consumption elevate oxidative stress, exacerbate lipid imbalances, and increase inflammation. Regular physical activity, with at least 150 min of moderate exercise per week, enhances insulin sensitivity and overall metabolic health. Quality sleep, averaging seven to nine hours per night, supports metabolic balance and weight control. Effective stress management strategies such as yoga, meditation, and therapy help reduce cortisol-related fat accumulation. Smoking cessation and moderation in alcohol intake lower inflammation and improve cardiovascular health [80,81,82,83].

It is our perspective that the prevalence of MetS is increasing due to factors such as urbanization, sedentary lifestyles, and the widespread consumption of processed foods. Genetic predisposition contributes to increased vulnerability among certain ethnic groups, while inconsistencies in diagnostic criteria may lead to an underestimation of its true prevalence. Socioeconomic disparities further exacerbate the risk, particularly among lower-income populations with limited access to healthcare and nutritious food. Emphasis should be placed on prevention rather than solely relying on medication, with a focus on education, dietary improvements, and physical activity. Additionally, the COVID-19 pandemic has intensified the burden of MetS by promoting sedentary behaviours and unhealthy eating habits. Effectively addressing MetS requires a comprehensive strategy that integrates lifestyle modifications, early detection, and policies aimed at ensuring equitable healthcare access.

### 3.3. Useful Indices in MetS

Several indices that have demonstrated utility in the assessment of MetS can be seen in Table 3.

### 3.4. Pathophysiology of MetS

The pathophysiology of MetS remains a topic of incomplete elucidation. However, there is ongoing discourse regarding the interplay between genetic and epigenetic elements, environmental factors, dietary habits, and lifestyle choices in the genesis of MetS [89,90]. Although the precise characterisation of MetS in the context of paediatric populations continues to provoke debate, it is unequivocal that IR linked to obesity constitutes the fundamental impetus behind its pathogenesis.

#### 3.4.1. Insulin Resistance and MetS

The study conducted by Barseem et al. (2015) highlights a substantial incidence of IR, affecting a notable 53% of obese children and adolescents. It is noteworthy that IR may precede and foster the onset of MetS in paediatric populations [91]. The accumulation of fatty acids within adipocytes, the liver, and skeletal muscles precipitates IR, exerting significant effects on glucose regulation and bolstering the transcription of genes responsible for hepatic lipogenic enzyme production [89]. In skeletal muscle, the pro-inflammatory state triggered by TNFα leads to diminished activity of AMP-activated protein kinase (AMPK) [92].

**Figure 2:**
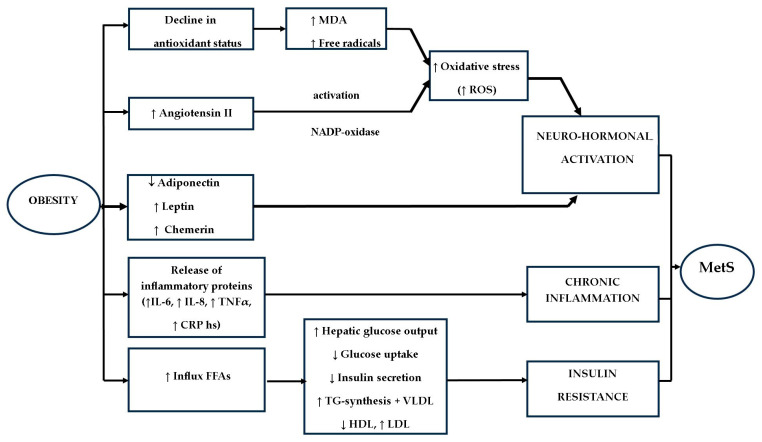
Pathophysiology of MetS (↑—increased, ↓—decreased).

Hepatic IR exerts an impact on insulin-mediated glucose homeostasis, influencing the augmented influx of free fatty acids (FFA). This, in turn, interferes with insulin’s hepatic function, thereby elevating hepatic glucose output and inhibiting glucose uptake via the modulation of protein kinase activation in muscle. Consequently, it results in an augmented activation of protein kinases in the liver, pro-inflammatory cytokine synthesis, elevated TGs, lowered HDL, and an upsurge in low-density lipoprotein cholesterol (LDL-C) [93,94]. As FFA is recognized as cytotoxic to beta-pancreatic cells, its increased presence culminates in reduced insulin secretion. The development of hypertension is attributed to the vasodilatory effects of insulin counteracted by the vasoconstrictive properties of FFA. Adipose tissue releases pro-inflammatory cytokines, amplifying the susceptibility to CVD. The augmented provision of FFA to the liver prompts heightened TG synthesis and the production of triglyceride-rich very low-density lipoprotein (VLDL) containing apolipoprotein B. Indirect consequences of IR encompass the depletion of HDL and the augmentation of LDL-cholesterol—Figure 2 [94].

IR also coincides with adipocyte dysfunction, typified by reduced adiponectin levels and heightened concentrations of FFA and TGs, thereby further elevating oxidative stress [95]. A negative correlation is established between increased insulin values and HDL levels, while positive correlations are evident with DBP, SBP, very low-density lipoprotein cholesterol (VLDL-C), LDL-cholesterol, total cholesterol, TGs, and glucose levels. A robust correlation is also observed between WC and triacylglycerol levels [96]. The presence of IR compounds the clearance of VLDL, resulting in the accumulation of lipoproteins, which fosters atheroma formation. Moreover, IR contributes to the emergence of a prothrombotic state, attributable to heightened serum viscosity [90]. The mechanisms underlying the elevation of blood pressure encompass renal sodium retention, heightened sympathetic nervous system activity, and smooth muscle proliferation [79,89].

Personal opinions on insulin resistance and MetS emphasize its multifactorial nature. Many view insulin resistance as the core driver of MetS, influenced by poor diet, sedentary lifestyles, and stress. Genetic predisposition and socioeconomic factors also play a significant role, with disparities in healthcare access exacerbating the condition. While medications can help, most advocate for prioritizing lifestyle interventions such as nutrition, exercise, and stress management. Additionally, chronic stress, hormonal imbalances, and sleep disorders are considered important contributors. A holistic approach, integrating prevention, early detection, and public health policies, is seen as essential for effectively addressing MetS.

#### 3.4.2. Obesity and MetS

It is well established that the predominant cause of IR in both children and adults is obesity. Specifically, in the context of obesity-related MetS, a defining characteristic is the accumulation of visceral (intra-abdominal) fat, indicative of central obesity, as well as the presence of ectopic adipose tissue in aberrant locations [97]. Adipose tissue serves as an endocrine organ, producing adipokines with varying pro- and anti-inflammatory properties, including but not limited to Interleukin-6 (IL-6), tumour necrosis factor-alpha (TNF α), leptin, adiponectin, resistin, chemerin, and visfatin—Figure 2 [89]. The pattern of fat storage in distinct depots, termed “lipid partitioning”, plays a pivotal role in shaping the profile of adipocytokines and their associated effects [93].

In children, the presence of obesity, particularly central obesity, exhibits a positive correlation with IR and is associated with elevated BP, decreased HDL levels, increased TG, and abnormal glucose regulation [98]. Excess adipose tissue leads to heightened lipolysis and free fatty acid (FFA) turnover, resulting in their release into the bloodstream. The infiltration of macrophages into adipose tissue prompts adipocyte hypertrophy and the release of cytokines that influence insulin function [93].

Central obesity, in particular, has been observed to induce MetS by modulating signalling pathways that govern metabolic equilibrium and energy homeostasis. Changes in body weight, indicative of an increasing body fat content, correlate inversely with blood sugar levels and insulin sensitivity [95]. Visceral fat, in comparison to subcutaneous fat, exhibits a stronger association with IR, partly attributed to its greater resistance to insulin action [94,99]. Furthermore, the deposition of fat within the skeletal muscle can impact the pathophysiology of MetS by affecting insulin signalling through the activation of protein PKCɛ induced by metabolites [93].

Obesity is also linked to subclinical chronic inflammation, characterised by the release of pro-inflammatory proteins into the circulation, including IL-6, TNF α, interleukin 8 (IL-8), and high-sensitivity C-reactive protein (C-RP hs). This inflammatory state interferes with insulin function, establishing a connection between obesity and IR, while initiating endothelial dysfunction and the early stages of atherosclerosis [92,100]. The production of IL-6 escalates with increasing body fat and the presence of IR, while elevated TNF α levels are associated with two key components of MetS: obesity and IR [94]. TNF α disrupts insulin-signalling adipocytes and hepatocytes, contributing to IR by inducing hepatic lipolysis. Heightened secretion of IL-6, TNF α, and C-RP is evident in individuals with abdominal obesity, intensifying the inflammatory state [99]. Toll-like receptors (TLRs) within the innate immune system contribute to the inflammatory state in MetS by activating inflammatory signalling pathways that foster the release of cytokines such as IL-6, TNF α, and IL-1 [90].

Personal opinions on obesity and MetS may vary. Many see obesity—especially visceral fat—as a key driver of MetS due to its role in insulin resistance and inflammation. However, some argue that not all obese individuals develop MetS, highlighting genetic, ethnic, and metabolic differences. Socioeconomic factors, such as limited access to healthy food and healthcare, also contribute to both conditions. While medications can help, most advocate for lifestyle interventions, including diet, exercise, and behavioral therapy. Psychological factors like stress and emotional eating are also considered important. A holistic approach that integrates prevention, lifestyle changes, and public health initiatives is widely supported.

#### 3.4.3. Oxidative Stress and MetS

Avelar et al. (2015) have noted the potential involvement of oxidative stress in the development of MetS, often associated with a decline in antioxidant defences [101]. Investigations focusing on malondialdehyde (MDA) levels have detected elevations in this marker and free radicals among obese individuals, suggesting that oxidative stress plays a role in the pathophysiology of MetS—Figure 2. Oxidative stress also exerts an impact on insulin sensitivity, thereby contributing to an increased risk of developing T2DM. Disruptions in the production of reactive oxygen species (ROS) escalate lipid peroxidation, promoting a pro-inflammatory milieu and raising the risk of atherosclerosis. An elevated concentration of nicotinamide adenine dinucleotide phosphate (NADPH) oxidase is further linked to oxidative stress and the risk of atherosclerosis, hyperglycemia, and the pro-inflammatory state associated with MetS, leading to its overactivation [101].

One of the three isoforms of the pleiotropic enzyme, paraoxonase 1 (PON1), demonstrates robust antioxidant activity and is tied to HDL, offering protection against oxidative stress and lipid peroxidation. It also enhances the antioxidant capacity of monocytes and macrophages. As observed by Senti et al. (2003), individuals with MetS exhibit significantly reduced levels of PON1, suggesting a correlation between the severity of MetS and a progressive deterioration in the antioxidant/peroxidant balance. The presence of multiple MetS components exacerbates oxidative stress [102].

Feoli et al. (2014) identified a noteworthy association between the activity of xanthine oxidase and the components of MetS, with this enzyme’s activity also correlating with C-reactive protein (C-RP) levels. Additionally, gamma-glutamyl transferase (GGT), indicative of oxidative stress, has potential as a diagnostic biomarker for MetS. Obesity exerts an influence on circulating GGT levels, which, in turn, correlates with various metabolic disorders such as hypertriglyceridemia, hypertension, and glucose abnormalities [103]. Nakanishi et al. (2004) suggested that GGT may reflect subclinical inflammation [104].

Yudkin et al. (2007) contend that inflammation, driven by the elevation of inflammatory factors, may underlie the development of IR [105]. Irrespective of the causal relationship, it is indisputable that both IR and inflammation are significant pathological pathways in the pathophysiology of MetS [13].

We consider that oxidative stress is a key factor in MetS, contributing to insulin resistance, inflammation, and endothelial dysfunction. Lifestyle and diet, particularly high consumption of processed foods, are seen as major contributors, while antioxidant-rich diets may help counteract oxidative damage. Obesity is linked to mitochondrial dysfunction and increased reactive oxygen species (ROS), making weight management essential. Genetic and environmental factors also influence oxidative stress levels, supporting personalized prevention strategies. While some advocate for antioxidant supplements, others prefer whole-food sources. Psychological stress further exacerbates oxidative stress, highlighting the importance of stress management. A holistic approach integrating lifestyle, diet, stress reduction, and targeted therapies is often recommended for managing MetS effectively.

#### 3.4.4. Adipocytokines and MetS

A central clinical repercussion of IR is the manifestation of dysfunctional adipose tissue, often referred to as “adiposopathy”, characterised by the emergence of hypertrophic adipose cells. Adipose tissue functions as a significant endocrine organ, secreting a myriad of factors collectively referred to as adipocytokines. Besides their established roles in various physiological processes such as growth, bone metabolism, reproduction, and immune responses, the involvement of adipocytokines has been evident not only in obesity but also in nonalcoholic fatty liver disease and MetS among children and adolescents [106,107].

The most extensively studied adipocytokines related to the pathogenesis of MetS include leptin, adiponectin, and more recently, chemerin and fibroblast growth factor 21, along with resistin and visfatin [3]. Leptin, a hormone involved in regulating food intake and lipid as well as carbohydrate metabolism, functions by curbing appetite and anabolic pathways while stimulating satiety and catabolic pathways. Resistance to leptin, particularly in skeletal muscles, has been linked to insulin resistance and the development of nonalcoholic fatty liver disease and MetS in children [107]. Additionally, leptin resistance may contribute to abnormal lipid partitioning within adipocytes [93]. Elevated leptin levels in obesity, coupled with reduced adiponectin levels in individuals with increased adipose tissue mass, correlate with an elevated cardiovascular risk [94].

A more robust predictor for MetS in both adults and children is adiponectin, known for its anti-atherogenic, anti-diabetogenic, anti-inflammatory, and anti-cell proliferation properties, offering protection against the onset of T2DM and CVD [108]. Low plasma adiponectin levels in children and adolescents have been associated with obesity, visceral adiposity, intramuscular fat, IR, T2DM, elevated blood pressure, and an atherogenic profile characterised by increased triglycerides, elevated apolipoprotein B levels, higher low-density lipoprotein (LDL) cholesterol, lower HDL cholesterol, and an augmented risk of malignancy—Figure 2. This condition triggers an inflammatory process within adipose tissue [93,99,100,109,110]. However, Jia et al. (2020) reported a negative correlation between childhood MetS Z-scores and adiponectin levels in adults, in contrast to the positive correlation with insulin levels [111]. Recent studies have introduced Fetuin-A, a liver-secreted protein, as a potential adipokine elevated in obese individuals, which may contribute to the onset of IR, as demonstrated in animal models [90].

In the context of MetS, the confluence of high leptin levels in obesity and low adiponectin levels in individuals with increased adipose tissue mass is associated with an elevated cardiovascular risk [94].

Another notable adipocytokine is chemerin, which is produced by adipocytes and plays roles in adipogenesis, inflammation, angiogenesis, as well as carbohydrate and lipid metabolism. While studies conducted on animal models have demonstrated these functions, research on human subjects, albeit on smaller cohorts, has revealed elevated chemerin levels, particularly in obese children with concurrent vitamin D deficiency [90,112].

Regulating adipogenesis, chemerin influences body weight and exhibits increased expression in white adipose tissue [113]. Substantial correlations have been established between chemerin and endothelial dysfunction, inflammation, and markers of metabolic and cardiovascular abnormalities [112]. Chu et al. (2012) have suggested that the combination of elevated chemerin levels with reduced adiponectin levels heightens the risk of developing MetS [114].

While the precise role of resistin in the pathogenesis of IR, T2DM, and MetS remains subject to debate, several studies have indicated its involvement in inflammatory processes and atherogenesis [100,115].

Steppan et al. (2001) have reported a positive association between resistin levels and various metabolic parameters, including WC, BP, blood glucose, cholesterol, very-low-density lipoprotein (VLDL), and insulin. They have also highlighted a positive correlation with inflammatory markers such as IL-6, TNF α, and C-RP [115].

Visfatin demonstrates a positive correlation with obesity and insulin resistance, with elevated levels observed in individuals with obesity and T2DM [113]. Fibroblast growth factor 21 primarily exerts antihyperglycemic and antihyperlipidemic effects. Animal studies have illustrated its capacity to promote lipolysis in white adipose tissue, enhance insulin signalling, facilitate liver glycogen production, and reduce gluconeogenesis [116]. Additional research suggests that various other adipocytokines, including retinol-binding protein 4 (RBP4), lipocalin-2, omentin-1, vaspina, and others, might serve as potential biomarkers for assessing metabolic disorders [107].

We belive that adipocytokines play a key role in MetS by regulating insulin sensitivity, inflammation, and lipid metabolism. An imbalance between pro-inflammatory and anti-inflammatory adipocytokines, often driven by obesity, contributes to MetS progression. Lifestyle factors like diet and exercise can help restore this balance, while emerging therapies targeting adipocytokines offer potential treatment options. Genetic and epigenetic factors also influence adipocytokine regulation, supporting personalized approaches. A holistic management strategy combining weight control, dietary improvements, physical activity, and stress reduction is widely recommended for MetS prevention and treatment.

#### 3.4.5. Renin-Angiotensin System and MetS

An essential neurohormonal pathway associated with the development of MetS is the Renin-Angiotensin System (RAS) [117]. RAS plays a contributory role in the emergence of MetS, with elevated production of Angiotensin II observed in individuals with obesity and IR—Figure 2. Angiotensin II, upon activation of nicotinamide adenine dinucleotide phosphate oxidase, leads to the generation of reactive oxygen species (ROS), which in turn catalyzes the oxidation of low-density lipoprotein (LDL). Additionally, cortisol, a stress-related mediator, promotes the accumulation of visceral fat. Notably, Wang et al. (2025) have documented positive associations between plasma corticosteroid levels and certains components of MetS [82].

#### 3.4.6. Epigenetics and MetS

The influence of epigenetics and developmental programming on MetS is a topic of discussion. Epigenetic mechanisms serve as the connection between programmed alterations in gene expression and exposure to environmental factors during pregnancy, impacting the growth and development of offspring [118]. Several studies have unveiled that epigenetic mechanisms are primarily responsible for triggering MetS in a majority of cases [119]. These epigenetic modifications are mediated by factors such as microRNAs, which have been recognized as biomarkers for various conditions, including metabolic disorders [120,121,122].

Research has demonstrated that microRNAs play a pivotal role in the pathogenesis of IR by influencing insulin secretion, biosynthesis, and the development of pancreatic β cells [123]. Furthermore, microRNAs have established associations with inflammation, endothelial dysfunction, alterations in lipid profiles, and other metabolic markers in children [124]. Additionally, the concept of gestational programming underscores that the hormonal, nutritional, and metabolic environment provided by the mother can exert a profound influence on the physiology and metabolism of the developing fetus [125].

#### 3.4.7. Gut Microbiota in MetS

The gut microbiome plays a critical role in metabolic regulation and the pathogenesis of MetS through its influence on nutrient absorption, immune modulation, inflammation, and host metabolism. Microbial imbalances can contribute to systemic inflammation, insulin resistance, and dyslipidemia, all of which are hallmarks of MetS. Emerging research continues to elucidate the complex interactions between gut microbiota and metabolic pathways, paving the way for microbiome-targeted interventions as a potential strategy for MetS prevention and management [126].

##### The Role of Gut Microbiota in MetS

The human gastrointestinal tract harbors a vast and diverse microbial ecosystem, fostering a symbiotic relationship with the host and significantly impacting both health and disease progression. Substantial research has underscored the intricate role of the gut microbiome in the development of MetS. A pivotal study by Vijay-Kumar et al. (2010) demonstrated that mice deficient in the Toll-like receptor 5 (TLR5) exhibited hallmark characteristics of MetS alongside an altered gut microbiome composition. Furthermore, transplantation of this microbiota into wild-type mice conferred several MetS-related traits, highlighting the microbiome’s influential role in metabolic dysregulation [127].

Various components of the gut microbiome contribute to maintaining gut barrier integrity, regulating inflammation, and influencing obesity, all of which are implicated in the onset of MetS. Early investigations into obesity-associated gut microbiome alterations revealed an increased *Firmicutes*-to-*Bacteroidetes* ratio. The higher metabolic diversity within *Firmicutes* compared to *Bacteroidetes* suggests an enhanced capacity for energy extraction from the diet. Notably, in vivo and in vitro studies indicate that *Bacteroidetes* predominantly reside in the luminal content, whereas *Firmicutes* tend to colonize the mucin layer [128].

*Firmicutes* bacteria indirectly interact with multiple tissues and organs via their metabolic byproducts, influencing appetite regulation and satiety. Conversely, *Bacteroidetes*, as gram-negative bacteria, exhibit immunomodulatory properties. A study by Carrizales-Sánchez investigating children (aged 7–17) with MetS and diabetes mellitus (DM) identified significant differences in gut microbiota composition at the family and genus levels. Specifically, *Faecalibacterium* and *Oscillospira* were more abundant in the MetS group compared to both DM patients and healthy controls, whereas *Ruminococcus* was notably reduced in DM patients. Furthermore, statistical analyses established positive correlations between cardiometabolic risk factors—including hypertension, abdominal obesity, hyperglycemia, and hypertriglyceridemia—and the genera *Prevotella*, *Dorea*, *Faecalibacterium*, and *Lactobacillus* [129].

**Figure 3:**
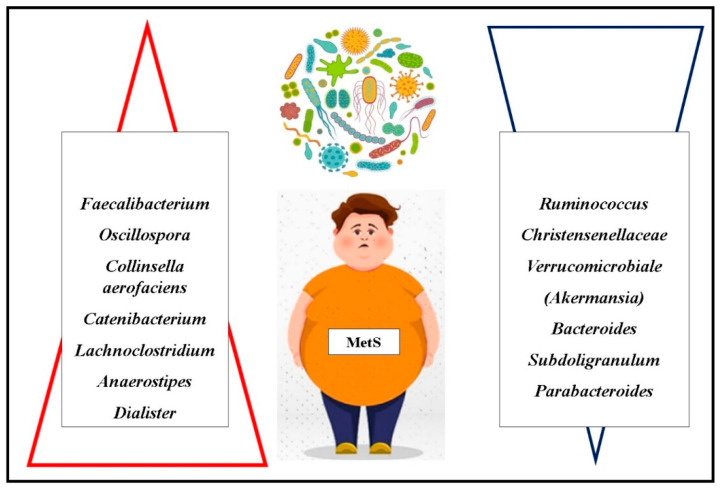
Gut Microbiota in MetS (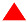
–increased;
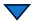
—decreased).

The prevalence of *Oscillospira* appears to be modulated by exogenous factors such as probiotic and prebiotic intake, natural dietary components, physical activity, and overall dietary patterns, as evidenced by Yang et al.’s research (Figure 3) [130].

##### Gut Microbiota and Its Mechanisms in Metabolic Regulation

The intestinal microbiota plays a crucial role in maintaining systemic homeostasis by influencing genes involved in energy metabolism. It affects appetite and satiety through vagus nerve activation and interactions with immunological and neuroendocrine pathways. Additionally, it contributes to bile acid metabolism and regulates hepatic triglycerides and glucose levels via the farnesoid X receptor. Moreover, the gut microbiota modulates the expression of the fasting-induced adipose factor (fiaf) gene, which enhances lipoprotein lipase activity, thereby facilitating lipid accumulation in adipose tissue. The microbiome also regulates systemic lipopolysaccharide (LPS) concentrations by maintaining epithelial barrier integrity. Disruptions in this regulation can lead to chronic low-grade inflammation, a condition frequently observed in MetS. A systematic review of human studies corroborates the association between elevated LPS levels and various metabolic syndrome components [131].

Chronic, low-grade inflammation, often triggered by nutrient excess, is a hallmark of obesity and a key driver of insulin resistance, thereby contributing to MetS pathogenesis. Within this framework, the *Parabacteroides* genus includes bacterial species that produce short-chain fatty acids (SCFAs), which are absorbed into the bloodstream via the intestinal epithelium. While SCFAs play a role in gut health, they can also disrupt lipid metabolism and contribute to metabolic dysfunction [132].

The primary SCFA-producing bacteria belong to the *Firmicutes* phylum, though butyrate production has also been reported in *Fusobacteria*, *Actinobacteria*, *Thermotogae*, and *Spirochetes*. Interestingly, despite not being natural butyrate producers, *Bifidobacterium* and *Lactobacillus* species can acquire this function through interactions with lactate- and acetate-producing commensals such as *Faecalibacterium*, *Eubacterium*, and *Roseburia* [131].

##### Microbial Diversity and Its Association with MetS

Alcázar et al. (2022) observed that children exhibiting MetS components had reduced microbial diversity, with a lower relative abundance of *Christensenellaceae* (*Christensenellaceae* R-7) and *Verrucomicrobiales* (*Akkermansia*) species compared to healthy controls. The potential link between *Akkermansia* and MetS is reinforced by the correlation between *Akkermansia muciniphila* and increased acetate production—an essential molecule in preventing weight gain. Acetate is known to stimulate the release of anorexigenic peptides and possesses anti-inflammatory properties [133].

Gallardo-Becerra et al. (2020) identified an elevated abundance of *Collinsella aerofaciens* (a *Coriobacteria* species) in children with obesity and MetS. This species showed a strong positive correlation with triglyceride and low-density lipoprotein (LDL) cholesterol levels. Furthermore, a species within the *Erysipelotrichaceae* family, *Catenibacterium*, was found in higher concentrations in individuals with obesity and MetS, suggesting its association with dyslipidemia and metabolic dysfunction [134].

A recent study by Wei et al. (2023) highlighted significant microbiome differences in children with MetS compared to obese but non-MetS controls. Specifically, *Lachnoclostridium*, *Anaerostipes*, and *Dialister* were enriched in the MetS group, while *Bacteroides*, *Subdoligranulum*, and *Parabacteroides* were depleted. These findings suggest that specific bacterial genera may serve as potential biomarkers for identifying obese children at risk for MetS. Moreover, the relative abundance of these genera was strongly associated with clinical indicators of MetS. Notably, *Lachnoclostridium* exhibited positive correlations with triglyceride levels, LDL cholesterol, and fasting blood glucose. This study also provided novel evidence suggesting that *Dialister* may have a negative impact on systolic blood pressure regulation [135].

### 3.5. Screening of MetS

A thorough assessment of children for behavioural and medical risks, particularly persistent obesity and its related co-morbidities, is essential [136]. Screening for MetS is recommended for overweight and obese children at increased risk for T2DM and CVD, especially when at least two of the following risk factors are present:

- Parental obesity
- Family history of T2DM (first or second-degree relatives)
- Racial/ethnic background (e.g., Native American, African American, Asian, Latino)
- Indicators of IR, such as acanthosis nigricans, HTN, PCOS, or dyslipidaemia
- A history of being small for gestational age
- Maternal history of gestational diabetes [137].

According to the American Diabetes Association (ADA), MetS screening should start in children over 10 or at puberty, with the Oral Glucose Tolerance Test (OGTT) as the gold standard, repeated every three years or FPG 100 mg/dL (5.6 mmol/L) to 125 mg/dL (6.9 mmol/L) Impaired Fasting Glycemia (IFG) or Glycated hemoglobin (A1C) 5.7–6.4% (39–47 mmol/mol [137].

Initial screening should involve a thorough history and physical exam to assess for comorbidities like PCOS, liver disease, and obstructive sleep apnoea, the latter diagnosable through polysomnography [138]. Overweight or obese children with additional risk factors should undergo biannual screening for liver damage, including serum alanine aminotransferase (ALAT) and aspartate aminotransferase (ASAT) levels; levels exceeding twice the normal range warrant hepatologist consultation [93,138,139].

For T2DM screening, overweight (≥85th percentile) or obese (≥95th percentile) children should be tested if they have at least one risk factor [137]. Annual blood pressure checks should start at age 3, with lipid screening beginning at age 9–11 [89,93]. Routine non-fasting, non-HDL lipid profile screening is recommended for children 9–11 years, while fasting lipid profiles should be conducted for 2–8-year-olds with moderate to high obesity risk [93].

## 4. Discussion

The rising prevalence of MetS in youth represents a critical yet often overlooked public health crisis. While obesity is widely acknowledged as a major risk factor for MetS, the intricate interplay between genetic predisposition, lifestyle choices, gut microbiota composition, and environmental influences requires further exploration. From our standpoint, addressing MetS in youth necessitates a multidisciplinary approach, integrating preventive strategies, early diagnosis, and targeted interventions.

One of the most pressing concerns is the lack of awareness among parents, educators, and healthcare providers regarding the long-term consequences of MetS in adolescents. The early onset of insulin resistance, dyslipidaemia, and hypertension significantly elevates the risk of premature cardiovascular disease and type 2 diabetes, yet routine screening for MetS is often absent in paediatric healthcare settings. In this context, we propose that universal screening protocols, especially in high-risk populations, could facilitate early intervention and reduce disease burden in adulthood.

A recurring theme in the literature is the potential reversibility of MetS through lifestyle modifications, yet real-world implementation remains a challenge. Encouraging children and adolescents to adopt healthier dietary patterns and engage in regular physical activity is an ideal solution, but adherence is often low due to socioeconomic barriers, digital screen dependency, and urbanization. Given the evidence linking gut microbiota dysbiosis to MetS, personalised dietary interventions—such as probiotic and prebiotic supplementation, fibre-rich diets, and reduced ultra-processed food consumption—warrant further research and clinical application.

The recent surge in microbiome research offers promising insights into novel therapeutic avenues for MetS. Given that specific bacterial taxa have been associated with insulin sensitivity, inflammation, and lipid metabolism, microbiota-targeted therapies, including faecal microbiota transplantation, next-generation probiotics, and microbiome-modulating drugs, could revolutionize MetS management. However, ethical concerns, safety profiles, and long-term effects of such interventions require extensive investigation before they can be widely adopted.

Despite the wealth of evidence linking obesity, gut microbiota, and MetS, a disconnection persists between research advancements and clinical translation. The lack of standardised diagnostic criteria for paediatric MetS further complicates early detection and intervention. Future research should prioritise longitudinal studies that explore the dynamic nature of MetS progression in youth, while policymakers must implement evidence-based nutritional policies, school-based wellness programs, and public health initiatives to curb the epidemic at its roots.

Ultimately, combating MetS in youth requires a paradigm shift in how we approach paediatric metabolic health. While genetic susceptibility plays a role, MetS is largely a preventable and manageable condition if addressed early. Thus, a synergistic approach combining lifestyle modifications, microbiome-targeted strategies, and healthcare policy reforms is paramount. As researchers and healthcare professionals, we must advocate for a proactive, rather than reactive, stance in tackling this growing epidemic before it manifests into an even greater burden on future generations.

### Research Gaps and Future Directions in Metabolic Syndrome: Paving the Way for Next-Generation Insights

MetS lacks a standardised definition, with various organisations applying differing diagnostic criteria, leading to inconsistencies in research outcomes and clinical management. While genetic and epigenetic factors influencing MetS are underexplored, further investigation into gene-environment interactions holds potential for novel insights. Although the gut microbiota’s role in MetS is gaining recognition, its therapeutic applications remain unclear. Inflammation and immune dysfunction are key contributors to MetS, yet their underlying mechanisms require additional study to inform the development of targeted anti-inflammatory therapies. The influence of sex, age, and hormones on MetS is insufficiently understood, underscoring the need for research aimed at tailored interventions. Although the link between MetS and mental health is acknowledged, further exploration of this bidirectional relationship is essential.

Pharmacological treatments for MetS currently lack specific, comprehensive therapies, and future drug development should prioritise multi-target, personalised approaches. The treatment of comorbidities in metabolic syndrome among children and a-dolescents is increasingly recognised as a critical area of research. The presence of multiple risk factors requires prompt therapy. Moreover, environmental and socioeconomic factors impact MetS, but the pathways involved are inadequately characterised. Lifecourse studies could identify early predictors of MetS, facilitating more effective prevention strategies. Interventions targeting modifiable biological and behavioural factors could provide insights into effective strategies for mitigating the impact of MetS in paediatric populations. These research gaps will pave the way for next-generation insights into the prevention and management of metabolic syndrome in children and adolescents. Additionally, the integration of digital health technologies, such as artificial intelligence and wearable devices, has the potential to transform the diagnosis, treatment, and monitoring of MetS. Addressing these research gaps is critical for advancing personalised, comprehensive management strategies for MetS.

## 5. Conclusions

Establishing global, consensus-driven criteria that account for ethnicity, gender, and age would enhance diagnostic accuracy and treatment approaches. The prevention of excessive weight among children and adolescents stands as a paramount objective within modern society’s healthcare system, demanding a multifaceted approach spanning individual, familial, institutional, communal, and public health level. The family physician and paediatricians play a pivotal role in identifying risk factors associated with excessive weight and instituting early interventions. Considering the complexity of the disease and the treatment, the team must be multidisciplinary and include a paediatrician, dietetician, kinesitherapist, diabetologist, cardiologist, psychologist, mental health practitioner, and nurses.

## Figures and Tables

**Table 1:** Comparison between different definitions of MetS proposed for children and adolescents.

Parameter	WHO, 1998 [2]	EGIR, 1999, Europe [19]	NCEPATPIII, 2001, America [20,21]	de Feranti, 2004, America [23]	IDF, 2007 [24]				IDEFICS, 2009, Europe [25]	Zong, 2022, International Definition [26]
Criteria of definition	IR + at least 2 of the following criteria		3 of the following criteria	≥3 of the following criteria	<6 years: there is no specific definition	6–10 years: not diagnosticatedccally	10–16 years: central adiposity + at least 2 of the following criteria	≥16 years: central obesity + at least 2 of the following criteria	2–9 years: HOMA-IR ≥ 90th percentile	6–17 years: abdominal obesity + at least 2 of the following criteria
WC	>95th percentile	≥90th percentile	≥90th percentile	≥75th percentile			≥90th percentile (values adjusted for sex and ethnicity)	Europe: >94 cm boys; >80 cm girls South/South-East Asian, Japanese, South/Central America: >90 cm boys; >80 cm girls	≥90th percentile	≥90th percentile for age and sex
BMI	>95th percentile									
BP			≥90th percentile for age, sex, height	>90th percentile			SBP ≥ 130 mmHg; DBP ≥ 85 mmHg	SBP ≥ 130 mmHg; DBP ≥ 85 mmHg	≥90th percentile	≥90th percentile for age, sex and height
TG (mg/dL)	>105–136 (<10 years)	≥150	≥110	≥100			≥150	≥150	≥90th percentile	≥100 (6–9 years old); ≥130 (10–17 years old)
HDL cholesterol (mg/dL)	<35 (>10 years)	≤40	≤40	≤50 (15–19 years of age)			<40	<40 boys; <50 girls	≤10th percentile	<40
Microalbu-minuria	present	excluded								
Glucose homeostasis		IFG/IGT	FPG ≥ 100 mg/dL or known T2DM	FPG ≥ 110 mg/dL			FPG ≥ 100 mg/dL or known T2DM	FPG ≥ 100 mg/dL or previously T2DM	FPG ≥ 90th percentile	FPG ≥ 100 mg/dL

**Table 2:** The prevalence of MetS.

Geographical Area	Country	Age of the Study Lot	Definition Criteria According Some Authors or Medical Associations	Prevalence of MetS	Reference
	Hungary	8–18 years	ATP III	8.9% in obese children	[37]
EUROPE	Romania	7–18 years	IDF 2009	55.8% in obese children	[38]
	Lithuania	10–17 years	IDF	Overweight: girls-8.7%, boys-20%	[39]
				Obese: girls-40.8%, boys-52.5%	
	Poland	10–12 years	IDF	girls-10.9%, boys-14.6%	[40]
	Slovakia	14–18 years	IDF	girls-0.4%, boys-2.7%	[41]
NORTH AMERICA	USA	≥12 years	ATPIII	non-Hispanic Whites-10.9%	[23]
				non-Hispanic Blacks-2.5%	
				Mexican Americans-12.9%	
	USA	12–20 years	Adolescent ATP Criteria	7.6%; no difference by gender	[42]
			IDF	9.6%; no difference by gender	
	USA	12–17 years	IDF	girls-2.1%, boys-6.7%	[43]
	USA	12–19 years	IDF	non-Hispanic Whites: girls-4.4%, boys-8.4%	[44]
				non-Hispanic Blacks: girls-4.2%, boys-2.5%	
				Mexican Americans: girls-6.4%, boys-9.4%	
	Canada	12–19 years	ATP III	3.50%	[45]
SOUTH AMERICA	Brasil	18–19 years	IDF	girls-6%, boys-3.7%	[46]
			de Ferranti et al.	girls-8.8%, boys-13.8	[23]
			Cook et al.	girls-2.8%, boys-6.3	[22]
	Mexico	8–15 years	ATP III	girls-21.2%, boys-23.1%	
			IDF	13.90%	[47]
	Peru	13–16 years	ATP III	girls-2.5%, boys-5.6%	[48]
			de Ferranti et al.	girls-14.4%, boys-19.9%	[23]
			IDF	girls-3.5%, boys-2.6%	
			WHO	girls-1.5%, boys-5.6%	
ASIA	Japan	6–19 years	NCEP-ATP III	17.7% in obese 6–11 years	[49]
				28.7% in obese 12–19 years	
	Turkey	7–18 years	WHO	7–11 years: 20%	
				12–18 years: 37.6%	[50]
				no difference by gender	
	Taiwan	6–12 years		girls-5.56%, boys-6.39%	[51]
	Iran	11–18 years	modified ATP III	girls-4.5%, boys-9.9%	[52]
	Korea	10–18 years	NCEP-ATP III	girls-5.5%, boys-5.8%	[53]
			IDF	girls-2.2%, boys-1.9%	
	China	7–18 years	IDF	girls-1.7%, boys-2.8%	[54]
	Eastern India	6–16 years	IDF	6–10 years: 11%	[55]
				11–16 years: 30.6%	
	South Korea	10–18 years	NCEP-ATP III	girls-3.4%, boys-4.8%	[56]
	India	10–19 years	NCEP-ATP III	girls-4.7%, boys-5.7%	[57]
AFRICA	Morocco	8–13.6 years	NCEP-ATP III	22%	[58]
	South Africa	13–18 years	NCEP-ATP III	girls-5.6%, boys-6.7%	[59]
	Tunisia	10–12 years	IDF	1% in overweight; 14.3% in obese	[60]
	Nigeria	10–19 years	NHLBI	girls-4.7%, boys-14.1%	[61]
	African countries	≤18 years	WHO	13.30%	[62]

**Table 3:** Useful indices in the assessment of MetS.

Index	Utility in the Assessment of MetS	Reference
Waist circumference (WC)	• a valuable indicator for evaluating abdominal obesity.	[84]
	• potential predictor of health risks associated with obesity and cardiovascular risk	
Body mass index (BMI)	• promising predictor for the development of MetS	[7]
Rohrer’s pondered index (PI)	• functions as a measure of obesity	[63,85]
	• a larger PI (calculated as weight/height³) at birth is associated with a rapid increase in BMI during childhood	
A body shape index (ABSI)	• an independent predictor of MetS, but with limited efficacy in identifying adolescents with MetS	[84]
	• correlates with the onset of MetS during adolescence	
Body round index (BRI)	• a novel non-invasive anthropometric index developed to predict body fat and the percentage of visceral adipose tissue	[84,86]
	• superior predictor for MetS when compared to BMI-z score and ABSI	
	• recognized as a MetS predictor	
Visceral adiposity index (VAI)	• associated with arterial stiffness, particularly in adults	[84,86]
Waist-to-height ratio (WHtR)	• exhibit enhanced predictive capabilities for MetS	[86]
Body composition	• analyses fat distribution • is essential for identifying at-risk youth	[10]
Bioelectrical impedance analysis (BIA)	• is a valuable and non-invasive tool, safe and effective for assessing body composition and visceral fat • is a reliable assessment tool for identifying individual at risk for MetS • aids in tailoring interventions for the vulnerable population • contributes to improving the management of obesity-related metabolic disorders	[87,88]

## Abbreviations

A1C	Glycated hemoglobin
ABSI	A body shape index
ADA	American Diabetes Association
AHA	American Heart Association
ALAT	alanine aminotransferase
AMPK	AMP-activated protein kinase
APOA1	apolipoprotein A1
ASAT	aspartate aminotransferase
BIA	Bioelectrical impedance analysis
BMI	Body Mass Index
BRI	Body round index
BP	blood presure
C-RP	C-reactive protein
CVD	cardiovascular disease
DBP	diastolic blood pressure
DHA	docohexaenoic acid
EGIR	European Group for the Study of Insulin Resistance
FPG	fasting plasma glucose
FFAs	free fatty acids
GGT	gamma-glutamyl transferase
HDL	high-density lipoproteins
HFpEF	preserved ejection fraction
HTN	hypertension
IDEFICS	Identification and Prevention of Dietary- and Lifestyle-Induced Health Effects in Children and Infants
IDF	International Diabetes Federation
IFG	Impaired Fasting Glycemia
IGT	Impaired Glucose Tolerance
IR	insulin resistance
LDL	low-density lipoproteins
LPS	lipopolysaccharide
MDA	malondialdehyde
MetS	Metabolic syndrome
NAFLD	non-alcoholic fatty liver disease
NCEP-ATP III	National Cholesterol Education Program—Adult Treatment Panel III
OGTT	Oral Glucose Tolerance Test
PON1	paraoxonase 1
PCOS	polycystic ovary syndrome
PI	ponderal index
RBP4	retinol-binding protein 4
RAS	renin-angiotensin system
SBP	systolic blood pressure
SCFAs	short-chain fatty acids
T2DM	type 2 diabetes melitus
TG	triglycerides
VLDL	very low-density lipoproteins
WHtR	Waist-to-height ratio
WC	waist circumference
WHO	World Health Organization
VAI	Visceral adiposity index
